# Full 360° Terahertz Dynamic Phase Modulation Based on Doubly Resonant Graphene–Metal Hybrid Metasurfaces

**DOI:** 10.3390/nano11113157

**Published:** 2021-11-22

**Authors:** Binxu Wang, Xiaoqing Luo, Yalin Lu, Guangyuan Li

**Affiliations:** 1 CAS Key Laboratory of Human–Machine Intelligence-Synergy Systems, Shenzhen Institute of Advanced Technology, Chinese Academy of Sciences, Shenzhen 518055, China; wangbx@siat.ac.cn (B.W.); xq.luo@siat.ac.cn (X.L.); 2 Nano Science and Technology Institute, University of Science and Technology of China, Suzhou 215123, China; 3 CAS Key Laboratory of Materials for Energy Conversion, Department of Materials Science and Engineering, University of Science and Technology of China, Hefei 230026, China; 4 Anhui Laboratory of Advanced Photon Science and Technology, University of Science and Technology of China, Hefei 230026, China; 5 Synergetic Innovation Center of Quantum Information & Quantum Physics, University of Science and Technology of China, Hefei 230026, China; 6 Hefei National Laboratory of Physical Science at the Microscale, University of Science and Technology of China, Hefei 230026, China; 7 Shenzhen College of Advanced Technology, University of Chinese Academy of Sciences, Shenzhen 518055, China

**Keywords:** graphene, metasurface, phase modulation, terahertz

## Abstract

Dynamic phase modulation is vital for tuneable focusing, beaming, polarisation conversion and holography. However, it remains challenging to achieve full 360° dynamic phase modulation while maintaining high reflectance or transmittance based on metamaterials or metasurfaces in the terahertz regime. Here, we propose a doubly resonant graphene–metal hybrid metasurface to address this challenge. Simulation results show that by varying the graphene Fermi energy, the proposed metasurface with two shifting resonances is capable of providing dynamic phase modulation covering a range of 361° while maintaining relatively high reflectance above 20% at 1.05 THz. Based on the phase profile design, dynamically tuneable beam steering and focusing were numerically demonstrated. We expect that this work will advance the engineering of graphene metasurfaces for the dynamic manipulation of terahertz waves.

## 1. Introduction

Metasurfaces have attracted increasing attention in the manipulation of the phase, amplitude, polarisation and wavefront of electromagnetic waves due to their appealing qualities including their compactness, easy integration and low loss [1,2,3,4]. Among these functionalities, the phase modulation is of particular importance [5] since it can lead to diverse relevant applications such as holographic imaging [6], polarisation manipulations [7], wavefront control [8,9], and absorbers [10,11]. Although various metasurfaces, either based on metals or based on all-dielectrics, have been designed or demonstrated to achieve full 360° phase modulations [1,12], most of these metasurface-based phase modulators are passive devices and cannot be actively tuned after fabrication.

Recently, great efforts have been dedicated towards active metasurface-based phase modulation [13,14,15,16,17] by incorporating graphene, stretchable substrates, or phase-change materials such as VO_2_ and chalcogenides. Since the first demonstration in 2009 [18], metasurface-based dynamic phase modulators have been of particular interest in the terahertz regime due to the lack of suitable natural materials. One design strategy is to make use of resonance frequency shifts induced by tuning the material’s properties. For example, Zhao et al. [19] demonstrated a large phase shift of up to 138° near 0.6 THz using a transmissive VO_2_ metasurface, however, the transmittance was only 2.5%. Nouman et al. [20] demonstrated reconfigurable terahertz phase control based on another VO_2_–metal metasurface, and realised a tuneable phase shift of 64° with a relatively high transmittance of 17%. Zhang et al. [21] demonstrated large terahertz dynamic phase modulation based on an enhanced resonant active HEMT metasurface, and achieved an experimental 137° dynamic phase shift, but with very low transmittance, only less than 5% (or less than −13 dB). On the other hand, based on a graphene–metal hybrid metasurface, Jung et al. [22] demonstrated terahertz phase modulators with a much higher transmittance of 53%. However, the dynamic phase shift is only 68°. Similarly, Hu et al. [23] made use of a silicon–metal hybrid metasurface and demonstrated ultrafast terahertz phase modulation with high transmittance of more than 25%. Cong et al. [24] also demonstrated a silicon–metal hybrid metasurface for ultrafast polarisation switching and dynamic beam splitting. However, the dynamic phase shift is also very limited (only 53°). Therefore, based on transmissive metasurfaces that make use of resonance frequency shifts via tuning the materials, there is a trade-off between the dynamic phase shift and the transmittance, and a large dynamic transmission phase shift above 180° has not been reported to date.

Another widely adopted design strategy for terahertz phase modulators is to make use of a reflective metasurface based on perfect absorption. For example, Miao et al. [25] demonstrated a wide-phase modulation range of 243° with gate-controlled reflective graphene metasurfaces. Liu and Bai [26] proposed a graphene metasurface and numerically obtained dynamic phase modulation of 180°. Based on graphene metasurfaces Kakenov et al. [27] and Tamagnone et al. [28], respectively, demonstrated a voltage-controlled terahertz phase modulation of *π*. Recently, Zhang et al. [29] proposed a graphene–metal hybrid metasurface and obtained dynamic phase modulation of up to 295° at a frequency of 4.5 THz. Although these reflective metasurfaces based on perfect absorption can achieve a much larger dynamic phase range than the transmissive metasurfaces based on resonance frequency shifts, the reflectance is very limited (usually less than 10%).

Therefore, based on the above two design strategies, it remains challenging to achieve a full 360° phase modulation while maintaining high transmittance/reflectance. However, in most applications such as tuneable metalens [30,31], beam steering [32,33], switchable wave-plates [34,35,36] and polarisation control [37,38], dynamic phase modulation covering the full 360° as well as high reflectance/transmittance are highly desirable. In order to tackle the challenge of the limited dynamic phase modulation range and relatively low reflectance/transmittance, Zhu et al. [39] proposed and demonstrated a multiple resonance metasurface for providing 360° phase variation in the microwave regime. Liu et al. [40] subsequently proposed a graphene metasurface composed of two resonators to achieve a dynamic 2*π* phase modulation and meanwhile, a high reflectance of 56% in the terahertz regime. Similarly, Ma et al. [41] also proposed stacked graphene metasurfaces and a numerically obtained dynamic reflection phase covering a range of nearly 2*π* while maintaining high reflectance in the far-infrared regime. Although these results are encouraging, the two closely packed graphene patch resonators of the terahertz metasurface unit cell in ref. [40] are isolated and thus it is difficult to tune the Fermi levels independently. To sum up, full 360° phase modulation is a fundamental and indispensable step for plenty of terahertz applications but one that remains challenging.

**Figure 1:**
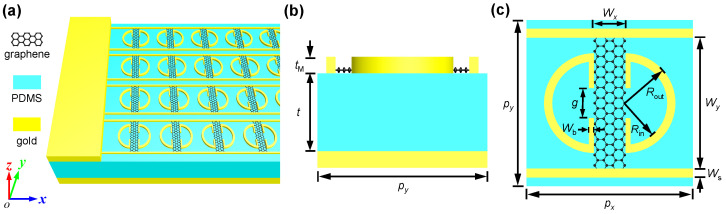
Schematic of the proposed metasurface, the unit cell of which is composed of gold stripes, gold asymmetric split rings and a graphene patch on top of a thick gold film sandwiched with a PDMS spacer: (**a**) 3D view; (**b**) side view; and (**c**) top view. $p_{x}=p_{y}=240$ μm; $W_{s}=4$ μm; $R_{\mathrm{out}}=90$ μm; $R_{\mathrm{in}}=75$ μm; $W_{b}=7.5$ μm; g=60 μm; $W_{x}=24$ μm; $W_{y}=228$ μm; $t_{M}=200$ nm; and t=45 μm.

In this work, we propose a graphene–metal hybrid metasurface based on double resonances in order to achieve full 360° dynamic phase modulation with relatively high reflectance—above 20% in the terahertz regime. The metasurface unit cell is composed of gold and graphene hybrid structures built on a reflective substrate sandwiched by a polydimethylsiloxane (PDMS) spacer layer. Distinct from the two closely packed graphene patch resonators in ref. [40], the graphene patches in this work are connected to the source/drain electrode via the gold stripes, facilitating the gate tuning of the Fermi levels of each row of graphene stripes, as illustrated in Figure 1. Simulation results will show that by tuning the graphene Fermi level, the metasurface exhibits two shifting resonances and dynamic 360° phase modulation with relatively high reflectance above 20% at the frequency of 1.05 THz. Based on the phase profile design, a terahertz meta-lens with tuneable focusing length and a dynamic beam deflector are numerically demonstrated.

## 2. Design and Methods

Figure 1 illustrates the proposed graphene–metal hybrid metasurface which is composed of two gold split-ring resonators (SRRs), two gold stripes and a graphene patch connecting them. The graphene–gold hybrid structures are built on top of a thick gold film sandwiched with a PDMS spacer layer. The graphene patches are connected to the source/drain electrode via the gold stripes and thus their Fermi levels can be dynamically tuned by gate voltages. The metasurface unit cell has periods of $p_{x}=p_{y}=240$ μm in both the *x* and *y* directions. The two parallel gold stripes have a width of $W_{s}=4$ μm and are separated by a distance of $W_{y}=228$ μm. The two gold SRRs separated by a gap width of $W_{x}=24$ μm have an outer radius of $R_{\mathrm{out}}=90$ μm, an inner radius of $R_{\mathrm{in}}=75$ μm and $W_{b}=7.5$ μm. The vertical bars of the SRRs are separated by a gap of g=60 μm. The graphene stripe connecting the gold stripes and SRRs has widths of $W_{x}=24$ μm and $W_{y}=228$ μm in the *x* and *y* directions, respectively. The gold stripes and SRRs have thickness of $t_{M}=200$ nm, and the PDMS spacer layer has a thickness of t=45 μm unless otherwise specified.

The proposed metasurface can be fabricated using state-of-art micro-fabrication processes. A thick gold film is first deposited onto a glass or silicon substrate. A PDMS layer is spin-coated on the top, followed by the transfer of a graphene layer. The graphene layer is then patterned using photolithography and plasma etching. Finally, the gold structures are patterned using photolithography and lift-off processes.

The proposed metasurface is illuminated by a normally incident terahertz plane wave with an electric field polarised along the *y* axis. The reflectance amplitude and phase spectra, as well as the near-field distributions were simulated using the frequency domain solver in CST Microwave Studio. Unit cells’ boundary conditions were adopted in both the *x* and *y* directions, and open boundary conditions were used in the *z* direction. The PDMS spacer was modelled with $\varepsilon_{r}$ = 2.35 and tanδ = 0.04. Gold was modelled using the lossy metal model with the electrical conductivity of $4.561\times 10^{7}$ S/m. The graphene surface conductivity σ can be decomposed into the interband conductivity $\sigma_{\mathrm{inter}}$ and the intraband conductivity $\sigma_{\mathrm{intra}}$. In the terahertz regime and at room temperature, the interband contribution can be safely neglected, and σ can thus be approximately expressed as [42,43]

(1) $$\begin{array}{c} \sigma \left(\omega \right)=\frac{ie^{2}E_{F}}{\pi \hbar^{2}\left(\omega +i/\tau \right)}. \end{array}$$

Here, *e* is electron charge, *ℏ* is the reduced Plank’s constant, $E_{F}$ is the Fermi energy level of graphene and τ is the transport relaxation time.

## 3. Results and Discussion

### 3.1. Phase Modulation Performance

**Figure 2:**
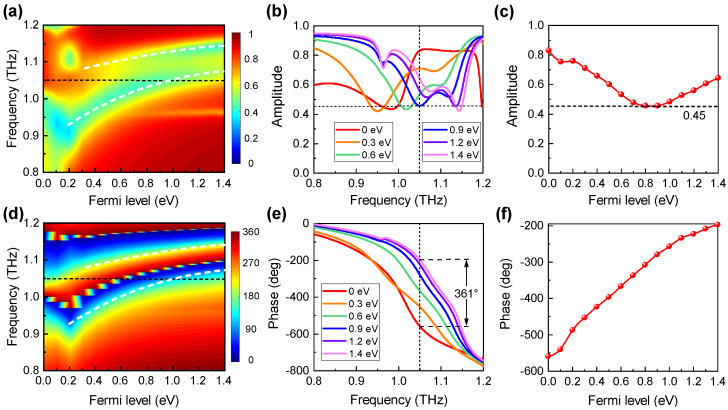
Simulated reflection (**a**,**b**) amplitude and (**d**,**e**) phase spectra for different graphene Fermi levels. Reflection (**c**) amplitude and (**f**) phase as functions of the graphene Fermi level at 1.05 THz.

Figure 2 depicts the dynamic reflection amplitude and phase spectra of the proposed metasurface with different Fermi levels of graphene. Figure 2a shows that for small graphene Fermi levels of $E_{F}\leq 0.4$ eV, there exists one dominant dip in the reflection spectra; whereas for larger graphene Fermi levels, an additional dip appears at the higher frequency. The frequencies of both reflection dips increase with the graphene Fermi level: as $E_{F}$ increases from 0 eV to 1.4 eV, the first dip frequency evolves from 0.93 THz to 1.08 THz, and the second evolves from 1.075 THz to 1.15 THz, as shown by Figure 2a,b.

The reflection phase spectra are shown in Figure 2d. It is clear that across each reflection dip frequency, the corresponding phase experiences a large shift of 360°. Therefore, at the frequency of 1.05 THz, close to which the two reflection dips appear for different Fermi levels, the reflection phase shift varies continuously, covering a large range of 361°, as the graphene Fermi level varies between 0 eV and 1.4 eV, as shown by Figure 2e. Figure 2f further shows that the phase increases almost linearly with the graphene Fermi level and the dynamic phase modulation reaches up to 361°. On the other hand, Figure 2c shows that the reflection amplitudes are always larger than 0.45, suggesting that the reflectance is above 20%. Correspondingly, the remaining energy (less than 80%) is absorbed by the metasurface since there is no transmission due to the metal mirror. We note that the dynamic phase modulation under *x*-polarised incidence has a much smaller range since the metasurface is polarisation-sensitive due to the anisotropic structure. Thus, the results under *x*-polarised incidence, which are of less interest, are not presented here for the sake of clarity.

**Figure 3:**
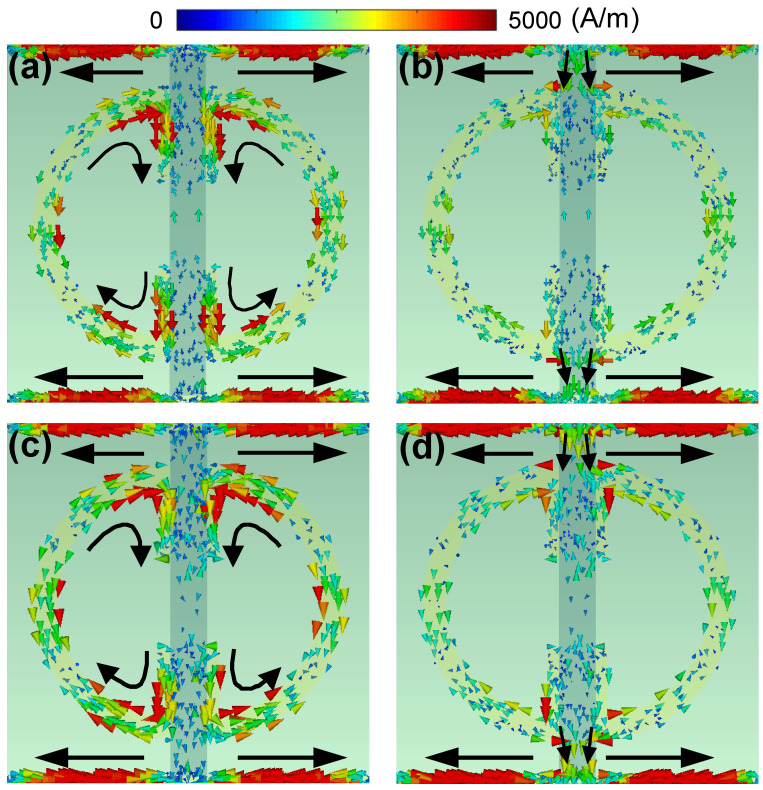
Surface current distributions of the metasurface with two typical graphene Fermi levels of (**a**,**b**) 0.6 eV for (**a**) 1.019 THz and (**b**) 1.1 THz and (**c**,**d**) 1.2 eV for (**c**) 1.068 THz and (**d**) 1.136 THz.

To investigate the two resonance characteristics of the proposed metasurface, in Figure 3, we plot the surface current maps for $E_{F}=0.6$ eV at 1.019 THz and 1.1 THz, and for $E_{F}=1.2$ eV at 1.068 THz and 1.136 THz. Given the graphene Fermi level, the two frequencies correspond to the two reflection dips. The results show that, for the first resonance at a relatively low frequency, the surface currents are mainly confined to the horizontal gold stripes and the SRRs; whereas for the second resonance at a relatively higher frequency, the surface currents are mainly confined to the horizontal gold stripes and the graphene patch. As the graphene Fermi level increases, the surface currents along the graphene patch become stronger.

**Figure 4:**
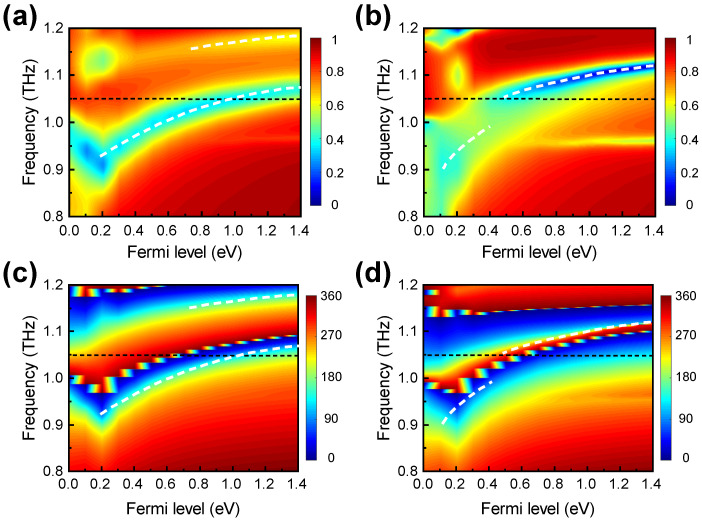
Simulated reflection (**a**,**b**) amplitude and (**c**,**d**) phase spectra for different graphene Fermi levels. The calculations were performed with (**a**,**c**) t=40 μm and (**b**,**d**) t=50 μm, respectively.

We emphasise that the geometric parameters adopted in this work were optimised. Among these parameters, the PDMS spacer thickness plays a vital role. Figure 4a shows that, for a smaller PDMS spacer thickness of t=40 μm, the spectral distance between the low-frequency resonance and the high-frequency resonance is enlarged. As a result, the dynamic phase modulation range at the frequency of 1.05 THz is only 310° when the graphene Fermi level varies between 0 eV and 1.4 eV, as shown by Figure 4c. In contrast, for a larger PDMS spacer thickness of t=50 μm, Figure 4b shows that the spectral distance between the low-frequency resonance and the high-frequency resonance is narrowed down. This leads to a much larger dynamic phase modulation range of 390° at a frequency of 1.05 THz, as shown by Figure 4d. On the other hand, Figure 4b shows that the corresponding reflection amplitudes are above 0.37, indicating that the minimum reflectance is only 13.7%.

### 3.2. Tuneable Beam Deflection

**Figure 5:**
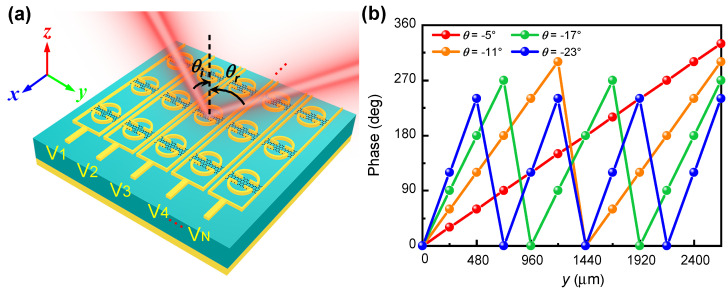
(**a**) Schematic of the phased array structure for achieving dynamic reflection beam steering; (**b**) reflection phase distributions of the designed phased array for achieving four specific deflection angles.

By dynamically controlling the graphene Fermi levels of the metasurface array, we can numerically realise a reflective phased array operating at 1.05 THz for concept proof, as illustrated by Figure 5. The metasurface array is composed of *N* rows of sub-array along the *y* axis, and the unit cell of each sub-array can be independently controlled using the gate voltage $V_{i}$, i=1,2,…,N. In the simulations, the time domain solver with the periodic boundary condition in both the *x* and *y* directions and open boundary condition in the *z* direction was employed. Under the incident terahertz plane wave polarised along the *y* axis, the anomalous reflection wavefront was deflected from the metasurface normal with a deflection angle of θ following the generalised Snell’s law Equation [44]:

(2) $$\begin{array}{c} \theta =\arcsin\left(\sin\theta_{\mathrm{inc}}-\frac{\lambda }{2\pi }\frac{\Delta \phi }{\Delta y}\right), \end{array}$$

where $\theta_{\mathrm{inc}}$ is the incident angle, λ is the operation wavelength, and Δϕ and Δy are the phase difference and the geometric distance between the neighbouring unit cells, respectively. As an example, we designed four deflection angles, $\theta =-5^{\circ }$, $-11^{\circ }$, $-17^{\circ }$ and $-23^{\circ }$, under normal incidence ($\theta_{\mathrm{inc}}=0^{\circ }$). The distance between two neighbouring unit cells is Δy=240 μm. According to Equation (2), the corresponding phase differences between the neighbouring unit cells should be Δϕ=π/6, π/3, π/2 and 2π/3, respectively. Thus, the theoretical numbers of unit cells in each sub-array are N=12, 6, 4 and 3, respectively. Figure 5b shows the required phase profile with $\phi_{i}=i\Delta \phi$ with i=1,2,…,N for the four designed deflection angles. As it is not practical to have smooth phase variations, we adopted discrete phase points to approximate the continuous phase profile. In simulations, the graphene Fermi levels corresponding to these reflection phase points can be determined by their quasi-linear relationship, as shown in Figure 2f.

**Figure 6:**
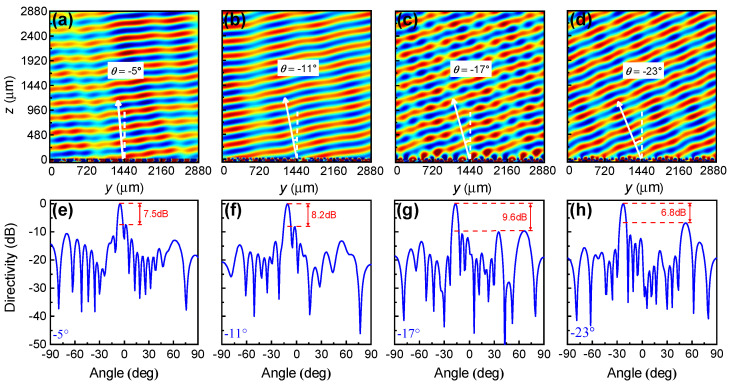
(**a**–**d**) Near-field electric field distributions $|E_{x}|$ and (**e**–**h**) far-field directivity for deflected reflection angles of (**a**,**e**) $\theta =-5^{\circ }$; (**b**,**f**) $-11^{\circ }$; (**c**,**g**) $-17^{\circ }$; and (**d**,**h**) $-23^{\circ }$.

Figure 6 depicts the simulated near-field electric field distributions and the far-field directivity for the designed four deflection angles. Results show that anomalous reflection angles of $-5^{\circ }$, $-11^{\circ }$, $-17^{\circ }$ and $-23^{\circ }$ can be clearly observed through the E-field pattern in Figure 6a–d, respectively. There are some distortions on the wavefront which correspond to sidelobes in the far-field directivity, as shown by Figure 6e–h. We note that the side lobes are almost random, which might introduce noises to the communication systems. We also found that the directivity differences between the main lobe and the side lobes for the designed four deflection angles reach 7.5 dB, 8.2 dB, 9.6 dB and 6.8 dB, respectively.

Note that, according to Equation (2), the signs of the deflection angles can also be flipped by reversing the phase point of each unit cell. Moreover, when Δϕ=0, which can be done by setting the same gate voltage or the same graphene Fermi level for all the unit cells, the reflection wavefront is not deflected, i.e., θ=0. For the sake of simplicity, these results are not shown here.

**Figure 7:**
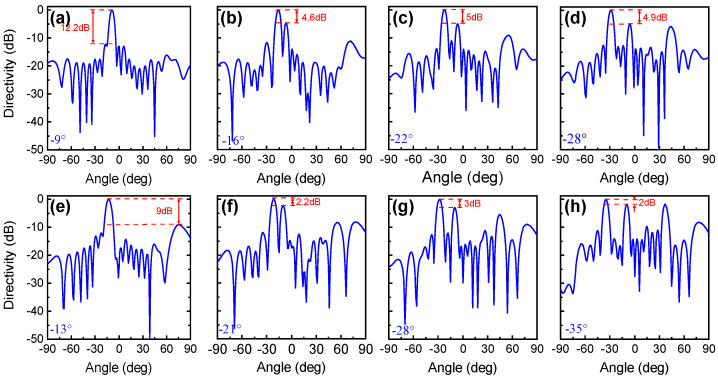
Far-field directivity for the deflected reflection angle of (**a**,**e**) $\theta =-5^{\circ }$; (**b**,**f**) $\theta =-11^{\circ }$; (**c**,**g**) $\theta =-17^{\circ }$; (**d**,**h**) $\theta =-23^{\circ }$ under oblique incident angles of (**a**–**d**) $5^{\circ }$; and (**e**–**f**) $10^{\circ }$.

We further illustrate the beam steering under the oblique incident angles of 5° and 10°, and plot the simulated far-field directivity in Figure 7. The results show that, as the incident angle increases, the reflected angle increases following Equation (2) and the difference between the main lobe and the largest sidelobe decreases: this difference is always greater than 3 dB for $\theta_{\mathrm{inc}}=5^{\circ }$ and larger than 2 dB for $\theta_{\mathrm{inc}}=10^{\circ }$. In other words, if the incident angle or the deflected angle increases, the directivity performance of beam-steering become worse.

### 3.3. Tuneable Focusing

**Figure 8:**
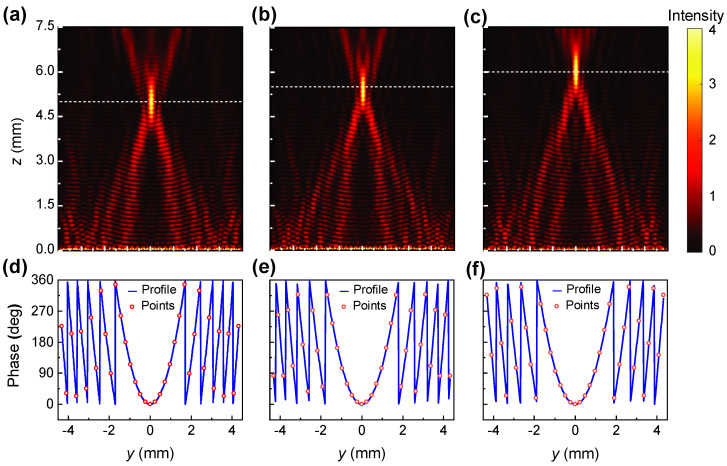
(**a**–**c**) Electric field intensity distributions $|E_{x}{|}^{2}$, (**d**–**f**) desired phase profiles (blue curves) and discrete phase points of the focusing metasurface array with designed focal lengths of (**a**,**d**) F=5 mm, (**b**,**e**) 5.5 mm and (**c**,**f**) 6 mm operating at 1.05 THz.

Given the same arrayed metasurfaces as illustrated by Figure 5a, we can also realise tuneable reflection focusing at 1.05 THz. As a proof of concept, we illustrate the tuning focusing with three different focal lengths of F=5 mm, 5.5 mm and 6 mm. For this purpose, the phase distribution of the array should be [1]:

(3) $$\begin{array}{c} \phi \left(y\right)=\frac{2\pi }{\lambda }\left(\sqrt{F^{2}+y^{2}}-F\right), \end{array}$$

where *F* is the designed focal length. The desired phase profiles calculated with Equation (3) for the three designed focal lengths at 1.05 THz are depicted by the blue curves in Figure 8d–f, respectively. In the simulations, each of these phase profiles are replaced by 37 discrete phase points that are given by

(4) $$\begin{array}{c} \phi \left(m\right)=\frac{2\pi }{\lambda }\left(\sqrt{F^{2}+{\left(md\right)}^{2}}-F\right), \end{array}$$

where y=md with m=0,±1,±2,…,±18 is incorporated for discretisation. In the simulations, the time domain solver with the periodic boundary condition in the *x* direction and open boundary condition in the *y* and *z* directions was adopted.

The red circles in Figure 8d–f show these discrete phase points. Figure 8a–c show a strong focusing effect of the reflected beams with the designed focal lengths of 5 mm, 5.5 mm and 6 mm can be observed, consistent with our expectation. The focused electric fields are greatly enhanced.

**Figure 9:**
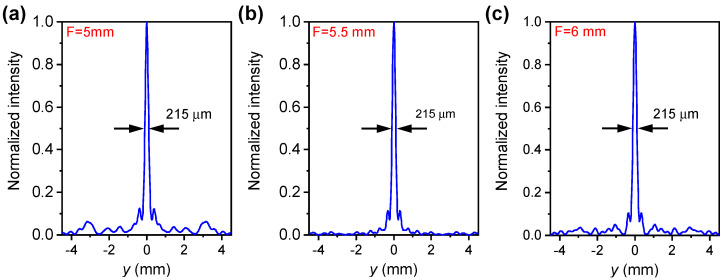
Normalised electric field intensity along *y* axis (when *z* locates at the focal points) for (**a**) F=5 mm, (**b**) 5.5 mm and (**c**) 6 mm, respectively.

To quantify the focusing quality, the electric field intensity along the *y* axis when *z* is fixed to the focal point is extracted and is shown in Figure 9. The calculated spot sizes, i.e., the full-width half-maxima (FWHMs), are all approximately equal to 215 μm. This corresponds to 0.75$\lambda_{0}$ for $\lambda_{0}=285.7$ μm (1.05 THz). Therefore, we find that the focusing intensities as well as the spot sizes are almost constant for these three different focal lengths. These characteristics are appealing in practical applications.

## 4. Conclusions

In conclusion, we numerically demonstrated full 360° dynamic phase modulation while maintaining relatively high reflectance above 20% in the terahertz regime based on a reflective graphene–metal hybrid metasurface with double resonances. The Fermi level of the graphene patch within the metasurface unit cell can be actively tuned by controlling the gate voltage. Results have shown that, at the frequency of 1.05 THz, around which two resonances can appear for different graphene Fermi levels, this remarkable dynamic phase modulation performance can be achieved. Based on the phase profile design, we further numerically demonstrated tuneable steering within the angle range of $\pm 23^{\circ }$, and a terahertz metalens with tuneable focal lengths within 5∼6 mm. Therefore, the presented graphene–metal hybrid metasurfaces could provide solutions to the active manipulation of terahertz waves in applications such as terahertz imaging, holography and telecommunications. As a final remark, we expect that the designed dynamic phase modulator can be extended to another frequency range by replacing the graphene with other two-dimensional materials [45].

## Data Availability

Data can be available upon request from the authors.
